# A 2010–2024 continuous GNSS dataset for Taiwan

**DOI:** 10.1038/s41597-026-07876-y

**Published:** 2026-07-15

**Authors:** Hsin Tung, Ya-Ju Hsu, Horng‐Yue Chen, Wen-Tzong Liang

**Affiliations:** Institute of Earth Sciences, Academia Sinica, Taipei, Taiwan

## Abstract

Continuous Global Navigation Satellite System observations provide important constraints on crustal deformation and a wide range of geophysical and environmental processes. Here we present a 15-year continuous GNSS dataset for Taiwan, comprising observations from more than 300 continuously operating stations distributed across the island and covering the period from 2010 to 2024. The dataset integrates observations from multiple agencies through a unified and internally consistent workflow that includes metadata handling, precise point positioning processing in the ITRF2020 reference frame, and systematic quality assessment, resulting in daily coordinate time series, station metadata, and associated quality metrics. Three key quality indicators, including data availability, satellite visibility, and the standard deviation of residuals from time-series modeling, are provided to support evaluation of station performance and the reliability of derived products. Instrumental and coseismic offsets are identified and recorded to support consistent and reproducible analyses. The dataset is archived in a version-controlled repository with complete metadata and detailed supporting documentation. This release represents a fixed dataset version to ensure reproducibility. The dataset provides a foundation for investigating crustal deformation, coseismic and postseismic deformation, hydrological loading effects, and other geodynamic processes in Taiwan.

## Background & Summary

**Fig. 1:**
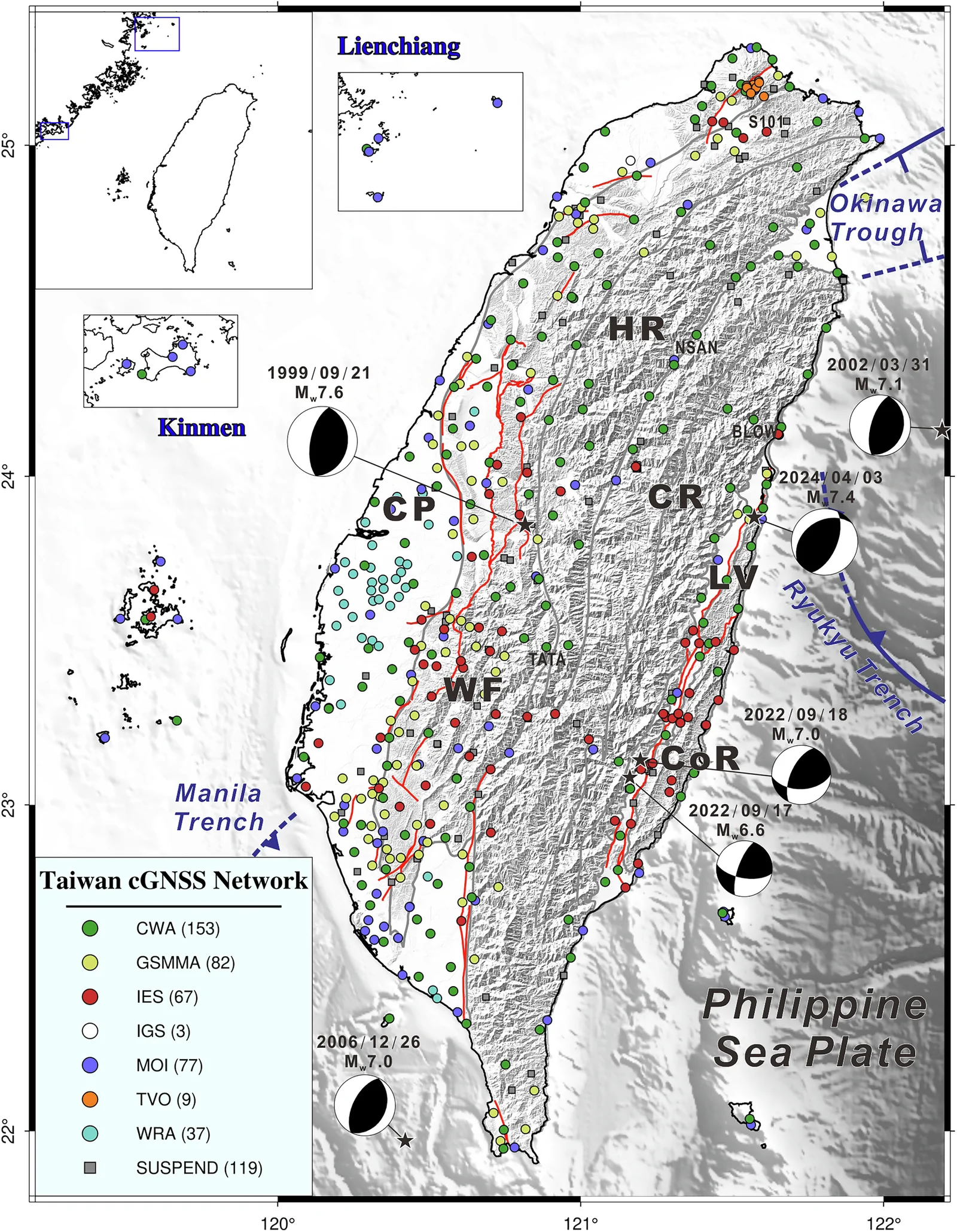
Taiwan cGNSS Network and locations of major M_w_ ≥ 7 earthquakes since 1990. Colored circles represent cGNSS stations operated by various agencies, including CWA (green), GSMMA (yellow), IES (red), MOI (purple), TVO (orange), and WRA (cyan). Grey squares indicate suspended stations. Red lines show active faults compiled by GSMMA. Focal mechanisms and epicenters of major M_w_ ≥ 7 earthquakes are labeled with dates and magnitudes. Insets show Kinmen and Lienchiang Islands near mainland China.

Taiwan lies at the convergent boundary between the Philippine Sea Plate and the Eurasian Plate, bounded by the Ryukyu and Manila subduction zones. This active tectonic setting results in frequent large earthquakes, rapid mountain uplift, and intense erosion^1–3^. Recent major earthquakes include the 1999 Mw 7.6 Chi-Chi event on the Chelungpu Fault^4–8^, the 2002 Mw 7.1 event near the southern Ryukyu Trench^9,10^, the 2006 Mw 7.0 Pingtung doublet^11–13^, the 2022 Mw 7.0 Chihshang sequence^14–16^, and the 2024 Mw 7.4 Hualien earthquake^17^ (Fig. 1).

Over the past three decades, space geodetic technologies, particularly the Global Navigation Satellite System (GNSS), have revolutionized the monitoring of surface deformation across multiple spatial and temporal scales. Taiwan’s cGNSS network, established in 1992, now comprises more than 500 stations operated collaboratively by several institutions, including the Institute of Earth Sciences, Academia Sinica (IES); the Central Weather Administration (CWA); the Mining Management Center of the Ministry of Economic Affairs (GSMMA); the Ministry of the Interior (MOI); the Taiwan Volcano Observatory (TVO); and the Water Resources Agency (WRA). At present, the network consists of over 500 stations, providing broad spatial coverage (Fig. 1).

The International GNSS Service (IGS)^18^ has demonstrated the importance of open data access and standardized processing strategies for achieving globally consistent geodetic products. Motivated by similar principles, continuous GNSS observations from Taiwan have been integrated into a unified and consistently processed dataset with harmonized metadata and systematic quality assessment procedures. Long-term GNSS observations constrain a wide range of deformation processes, including interseismic strain accumulation^19–22^, coseismic displacements^23–25^, postseismic relaxation^26–29^, and fault creep^30,31^, as well as environmental processes such as landslides, hydrological loading, sediment transport, and anthropogenic processes^32–43^.

Here we present continuous GNSS observations collected across Taiwan over a 15-year period from 2010 to 2024. The dataset is processed using a consistent precise point positioning (PPP) strategy in the ITRF2020 reference frame together with harmonized metadata management and quality assessment procedures. We provide daily coordinate time series, station metadata, and associated quality metrics to facilitate evaluation of station performance, data quality, and the reliability of derived geodetic analyses. The dataset serves as a resource for interseismic deformation studies, slow slip event detection, coseismic and postseismic deformation analyses, and investigations of hydrological and environmental influences.

## Methods

### Taiwan continuous GNSS array

The Taiwan continuous GNSS (cGNSS) network was established in 1992 and expanded rapidly following the 1999 Mw 7.6 Chi-Chi earthquake, supported by the National Science Council’s five-year Program for Earthquake and Active-Fault Research (PEAR). This initiative promoted rapid densification of the network, particularly in regions near active faults and areas critical to seismic hazards assessment.

**Fig. 2:**
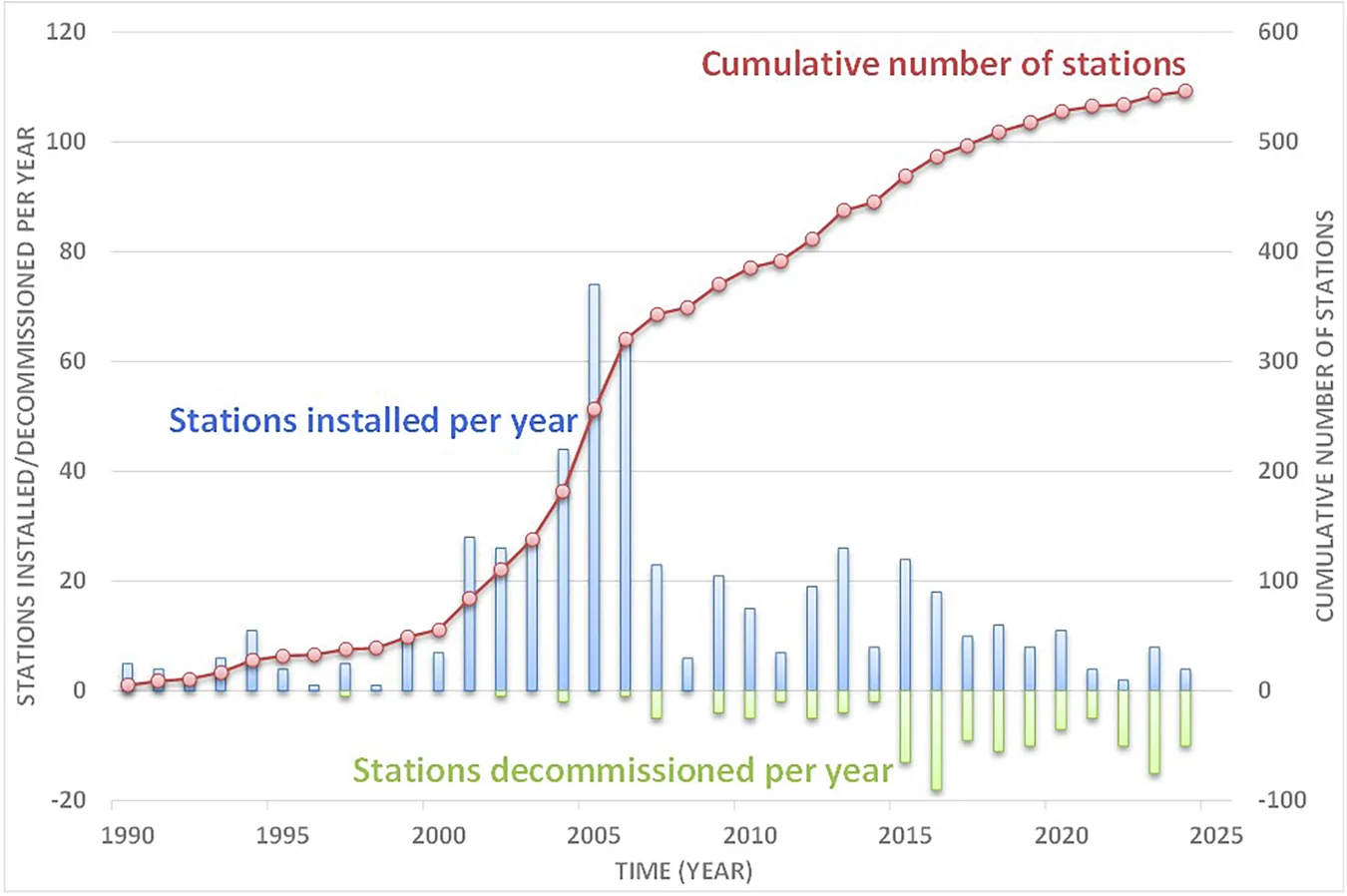
The annual number of installed and decommissioned stations and cumulative number of continuous GNSS stations across Taiwan from 1990 to 2024. Blue and green bars show the numbers of stations installed and decommissioned each year, respectively. Red dots indicate the cumulative number of stations.

By the end of 2005, collaboration between the CWA and the IES had established 160 new cGNSS stations and integrated approximately 50 existing sites into a unified network. Additional stations were subsequently contributed by other agencies, including the MOI, the GSMMA, and the WRA. The network expanded rapidly between 2004 and 2007, exceeding 350 stations by 2007, and has continued to grow at a rate of approximately 10–20 stations per year. By the end of 2024, the network comprised 537 stations (Fig. 2), of which approximately 430 remained in continuous operation after relocation or decommissioning of low-visibility sites. Participating agencies provide GNSS observations on a monthly basis. Before integration, the data undergo quality-control procedures to verify file completeness, format consistency, and metadata accuracy, including antenna and receiver information. All observations are standardized to the Receiver Independent Exchange Format (RINEX) for subsequent GNSS processing.

Although most receivers support multi-constellation tracking, the current dataset focuses exclusively on GPS observations because of software and format limitations. Accordingly, “GPS” refers specifically to processed observations and derived products, whereas “GNSS” refers more broadly to the multi-constellation instrumentation and observational infrastructure.

### GPS precise point positioning

All GPS observations were processed using the precise point positioning ambiguity resolution (PPP-AR) technique implemented in the GipsyX/RTGx software package^44^. Daily station coordinates were estimated from undifferenced dual-frequency GPS observations using final orbit and clock products provided by the Jet Propulsion Laboratory (JPL), together with ambiguity-resolution procedures based on JPL wide-lane phase-bias products. All solutions are expressed in the ITRF2020 reference frame.

The processing strategy used ionosphere-free linear combinations of dual-frequency GPS code and phase observations, an elevation cutoff angle of 10°, and a 300 s sampling interval. Zenith wet delay parameters and horizontal tropospheric gradients were estimated as stochastic parameters in the PPP filter. Tropospheric parameters, including zenith wet delay and horizontal gradients, were estimated as stochastic parameters in the PPP filter. VMF1-based tropospheric input files were generated for each station and used in the processing workflow^45^. Antenna phase-center variations were corrected using calibration models provided by the NOAA National Geodetic Survey^46^. Ocean tide loading corrections were applied using FES2004-based loading coefficients obtained from the Onsala Space Observatory ocean tide loading service^47,48^.

The processing was designed to provide a unified and internally consistent PPP-AR solution for the Taiwan cGNSS network in the ITRF2020 reference frame. The tropospheric and ocean-loading models used in this release, VMF1 and FES2004, were consistently implemented within our GipsyX/RTGx processing environment. Although more recent IGS repro3-oriented analyses may use updated models such as VMF3/GPT3 and FES2014b, the model choices adopted here provide a consistent basis for generating long-term daily coordinate time series and station-level quality metrics. Users requiring strict consistency with IGS repro3 products should consider these processing-model differences when making direct comparisons.

While many stations support multi-constellation tracking, this dataset incorporates GPS observations exclusively, thereby excluding inter-system scale differences introduced by mixed-constellation processing. Station coordinates were independently estimated using JPL products referenced to ITRF2020; no additional regional network adjustment was applied.

### GPS time series

GPS position time series contain signals associated with secular trends, seasonal variations, coseismic and instrumental offsets, postseismic relaxation, and transient deformation. Least-squares regression was applied independently to each coordinate component using the following model:

1 $$\begin{array}{c}y({t}_{i})=a+b{t}_{i}+c\cos (2\pi {t}_{i})+d\,\sin (2\pi {t}_{i})+e\,\cos (4\pi {t}_{i})+f\sin (4\pi {t}_{i})\\ \,\,\,\,+\,\mathop{\sum }\limits_{j=1}^{{n}_{g}}{g}_{j}\cdot H({t}_{i}-{T}_{gj})+\mathop{\sum }\limits_{j=1}^{{n}_{k}}{k}_{j}\cdot (1-{e}^{-\frac{({t}_{i}-{T}_{kj})}{{\tau }_{j}}})\cdot H({t}_{i}-{T}_{kj})+{v}_{i}\end{array}$$

where *a* and *b* represent the initial position and secular velocity, *c, d, e, f* denote annual and semi-annual amplitudes, *H* is the Heaviside step function for offsets of magnitude *g*_*j*_ at epochs *T*_*gj*_, and *k*_*j*_ and *τ* describe postseismic relaxation parameters. The term *v*_i_ denotes residual errors. This model was used to separate long-term trends, seasonal signals, and offset-related discontinuities within the time series.

Instrument-related offsets were identified using records of antenna or receiver changes provided by participating agencies. Coseismic displacements were screened using the CWA earthquake catalog^49^ based on earthquake magnitude and focal depth. We routinely examine events with local magnitude (ML) ≥6.0 and focal depth ≤35 km. Offshore earthquakes with ML ≥6.5 and depths up to 50 km were also evaluated when surface displacements were anticipated. Time-series segments exhibiting displacements exceeding two times the standard deviation were automatically flagged and subsequently verified through visual inspection to reduce false detections while maintaining sensitivity to deformation signals.

In addition to documented offsets, abrupt unmodeled changes were identified through manual inspection of the time series. Possible causes include localized ground deformation, undocumented maintenance, nearby construction activity, or temporary degradation of signal quality. These unmodeled changes are recorded in the dataset metadata to facilitate future reevaluation and interpretation. All detected offsets and discontinuities are annotated within the metadata accompanying the released dataset.

## Data Records

The GNSS dataset described in this study has been deposited in Zenodo as a fixed, version-controlled release, version 1, covering the period 2010–2024^50^. The archive provides consistently processed continuous GNSS products from more than 300 cGNSS stations across Taiwan and is organized into several components, including station metadata, quality metrics, processed coordinate time series, and associated documentation.

- **Station metadata**
  The archived dataset includes several metadata files documenting station coordinates, operating agency, installation date, antenna type, radome information, and receiver specifications (station_metadata.csv); detected coseismic offset timings (coseismic_displacements.csv); equipment-related discontinuity timings (antenna_or_receiver_changes.csv); and manually identified unmodeled discontinuities (abrupt_unmodeled_changes.csv) derived from the time-series analysis to facilitate interpretation and future reevaluation.
- **Quality information**
  The quality_metrics.csv file provides station-level quality indicators, including data availability, mean satellite visibility, and the standard deviation of residuals (SDR) for the east, north, and vertical components (SDR_E, SDR_N, and SDR_U), enabling objective evaluation of station performance and data quality. Example fields include station name, data availability (%), mean satellite visibility (%), and SDR values (mm) for each coordinate component.
- **Processed time series**
  The archive provides both primary daily PPP coordinate solutions and derived time-series products. The primary daily PPP coordinate solutions are provided before offset correction and seasonal filtering, except for the removal of clearly invalid daily solutions identified during quality control. These primary files allow users to apply their own time-series analysis strategies, including independent outlier screening, offset detection, stochastic noise modelling, and velocity estimation. The derived products include offset-corrected displacement time series accounting for coseismic and instrumental offsets, and seasonally filtered displacement time series after removal of annual and semi-annual signals. These derived products are provided as reproducible reference products generated using the procedures described in the Methods section. Users are therefore not required to adopt the time-series modelling strategy used in this study.
  Time series are provided in two complementary formats. The first format consists of original PPP solution files exported from the GipsyX/RTGx workflow, which contain daily coordinate solutions and associated processing information. The second format consists of simplified local east, north, and vertical displacement files, which provide decimal year, calendar date, east, north, and vertical displacements in millimeters, and the corresponding uncertainties for each component. Detailed file names, extensions, and column definitions are provided in the accompanying README file.

## Technical Validation

To evaluate the reliability, internal consistency, and long-term stability of the archived GNSS dataset, we conducted a series of quantitative assessments focusing on data continuity, satellite visibility, and the stability of position time series. These validation procedures provide objective indicators for evaluating station performance and identifying periods of reduced data quality. The assessments were applied to 328 stations installed prior to 2015 that remained in continuous operation throughout the evaluation period, ensuring sufficiently long observation records for robust estimation of long-term quality indicators.

### Availability of GNSS observations

Availability of continuous GNSS observations serves as a critical measure of time-series integrity, since data gaps can hinder reliable detection of short-term slip events. For each station, data availability was calculated as the ratio of valid daily observations to the total expected number of observation days during the evaluation period.

Among the 328 evaluated stations, 268 stations (82%) achieved data availability exceeding 90%, indicating high temporal continuity across most of the network. In contrast, 21 stations (6%) exhibited data availability below 75%. These lower-availability stations were excluded from the subsequent satellite visibility analysis and are identified in bold in quality_metrics.csv.

### Satellite visibility

Satellite visibility was evaluated as an indicator of local environmental conditions affecting GNSS signal reception. Using monthly observation files from 2015 to 2024, the number of satellites recorded at each epoch was compared with the expected number of visible GPS satellites derived from satellite geometry. The expected number of visible GPS satellites was estimated from daily broadcast navigation files (BRDC) obtained from CDDIS, using each station coordinate and a 10° elevation cutoff angle. For each epoch, satellites predicted to be above the cutoff angle were counted as expected satellites. This expected number was then compared with the number of satellites actually recorded in the corresponding observation file to compute the visibility ratio. The resulting visibility ratio quantifies signal obstruction caused by surrounding topography, vegetation, or nearby structures.

Among the 307 stations analyzed, 242 stations (approximately 80%) exhibited average visibility ratios exceeding 90%, consistent with installation practices that prioritize open-sky environments. However, visibility ratios alone do not directly represent positioning accuracy and should instead be interpreted as indicators of local sky visibility conditions. Several stations, including BLOW, TATA, and NSAN (locations shown in Fig. 1), exhibited lower visibility ratios of 68–79% because of persistent topographic obstruction in mountainous terrain. Because these obstructions remain stable through time, their influence on long-term velocity estimates is considered negligible.

Moreover, temporal variations in satellite visibility provide a more effective indicator of environmental changes that may influence GNSS data quality. In Taiwan’s subtropical maritime climate, dense vegetation and seasonal growth may partially obstruct satellite signals and increase multipath effects, potentially degrading positioning performance. Several stations exhibit gradual reductions in visibility over time followed by recovery after site maintenance or vegetation removal, highlighting the importance of monitoring temporal visibility changes when evaluating station conditions.

To systematically evaluate temporal visibility variations, monthly visibility time series from 2015 to 2024 were analyzed using two complementary approaches designed to identify periods of potential signal degradation.

1. Short-term visibility degradation
  For each station, a normalized relative visibility index *V(t)* was defined as:
  2 $$V(t)=\frac{R(t)}{{P}_{95}},R(t)=\frac{{N}_{obs}(t)}{{N}_{\exp }(t)}$$
  where *N*_*obs*_*(t)* and *N*_*exp*_*(t)* denote the numbers of observed and expected satellites at time *t*, respectively, and *P*_*95*_ represent the 95th percentile of the visibility ratio *R(t)* for each station during 2015 to 2024. This normalization reduces the influence of permanent site-specific obstruction, such as surrounding terrain, and enables consistent thresholding across the network. A degradation interval was identified when *V(t)* remained below a station-specific threshold ratio (0.97–0.98 depending on local environmental conditions) for at least three consecutive months, indicating sustained degradation relative to the station’s typical visibility conditions.
2. Long-term visibility degradation
  To identify persistent long-term degradation trends, an 18-month moving-window linear regression of monthly visibility values (in percent) was performed for each station. The resulting slope was expressed in percentage points per month (% month^−1^). A degradation episode was identified when the regression slope remained below −0.15% month^−1^ for at least four consecutive monthly estimates. This empirically defined threshold captures sustained visibility degradation while minimizing spurious detections caused by short-term fluctuations. This approach reduces the influence of transient visibility variations associated with seasonal vegetation growth or temporary environmental effects and provides a more stable indicator of long-term changes in station conditions.
  The two diagnostic approaches collectively capture both short-term visibility loss and long-term degradation trends. To avoid artificially separating prolonged degradation into multiple short intervals, any two degraded intervals separated by less than six months were merged into a single episode. This procedure minimizes the influence of temporary recovery periods that do not reflect genuine improvements in station conditions.

**Fig. 3:**
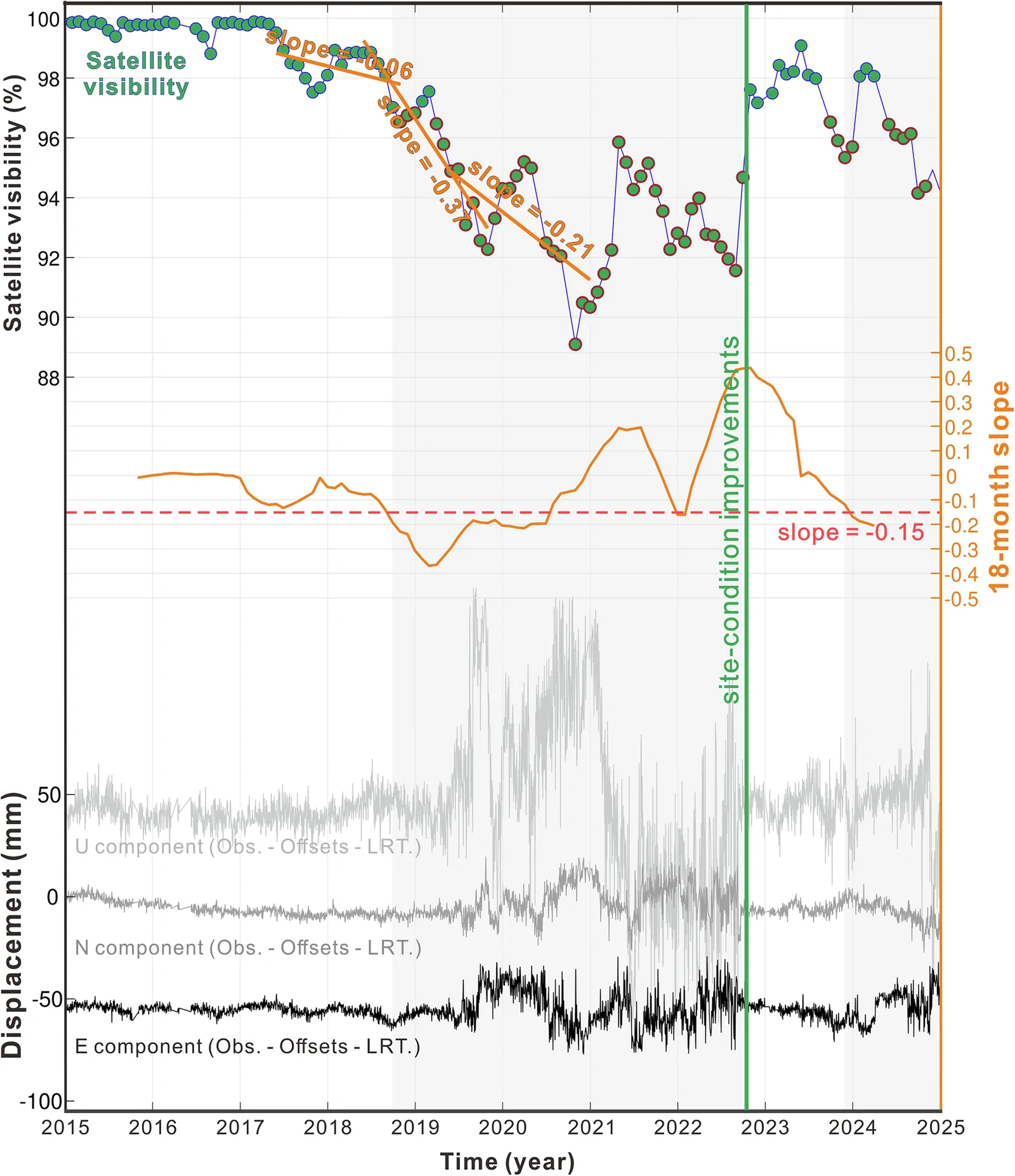
Representative example illustrating satellite visibility variations and associated quality indicators. Green circles represent monthly satellite visibility, and orange curve shows the slope estimated using 18-month moving-window visibility. Dashed red line indicates the degradation threshold of −0.15% month^−1^. Grey shaded regions indicate intervals classified as degraded visibility periods based on the criteria described in the manuscript. The vertical green line marks a period of site-condition improvement associated with recovery in satellite visibility. Background traces show residual GPS displacements in the east, north, and vertical components after removing secular trends and documented offsets.

Figure 3 illustrates representative examples of monthly satellite visibility, long-term visibility trends, and shaded intervals identified as degraded visibility periods according to the criteria described above. Residual GPS displacement time series in the east, north, and vertical components are also shown after removing secular trends and documented offsets.

Although the diagnostic thresholds are broadly applicable across the network, site-specific factors such as maintenance activities, vegetation trimming, antenna replacement, or firmware updates may also influence visibility ratios. These effects are generally minor compared with persistent environmental obstructions but are noted for completeness. Final degradation classifications were therefore manually reviewed and adjusted when necessary. Periods of degraded satellite visibility identified for each station are summarized in Supplementary Table S1 and provide a practical reference for evaluating station reliability and prioritizing future site inspections. Complete station-level quality metrics are provided in quality_metrics.csv within the archived dataset.

### Standard deviations of residuals from the least-squares regression

The third criterion used to evaluate GNSS data quality is the SDR after removing secular trends, annual and semi-annual seasonal signals, earthquake-related deformation, and instrumental offsets using the least-squares regression model described in the Methods section (Eq. 1). The SDR quantifies the level of scatter remaining in the time series and is defined as:

3 $$SDR=\sqrt{\frac{1}{n-p}\mathop{\sum }\limits_{i=1}^{n}{({y}_{i}-{\hat{y}}_{i})}^{2}}$$

where *n* is the number of observations, *p* is the number of estimated regression parameters, *y*_*i*_ is the observed position at epoch *i*, and ${\hat{y}}_{i}$ is the corresponding model prediction. Lower SDR values indicate stable site conditions and limited unmodeled variability, whereas elevated SDR values reflect increased scatter within the observations.

The SDR was computed independently for the east (E), north (N), and vertical (U) displacement components for each GNSS station, resulting in one SDR value per station and per component. The collection of SDR values from all stations was then analyzed to evaluate overall network data quality. The horizontal components generally exhibit low scatter, with network-wide median SDR values of 2.3 mm and 2.1 mm for the east and north components, respectively. In contrast, the vertical component exhibits substantially larger scatter, with a network-wide median SDR value of 6.5 mm and several stations exceeding 25 mm.

**Fig. 4:**
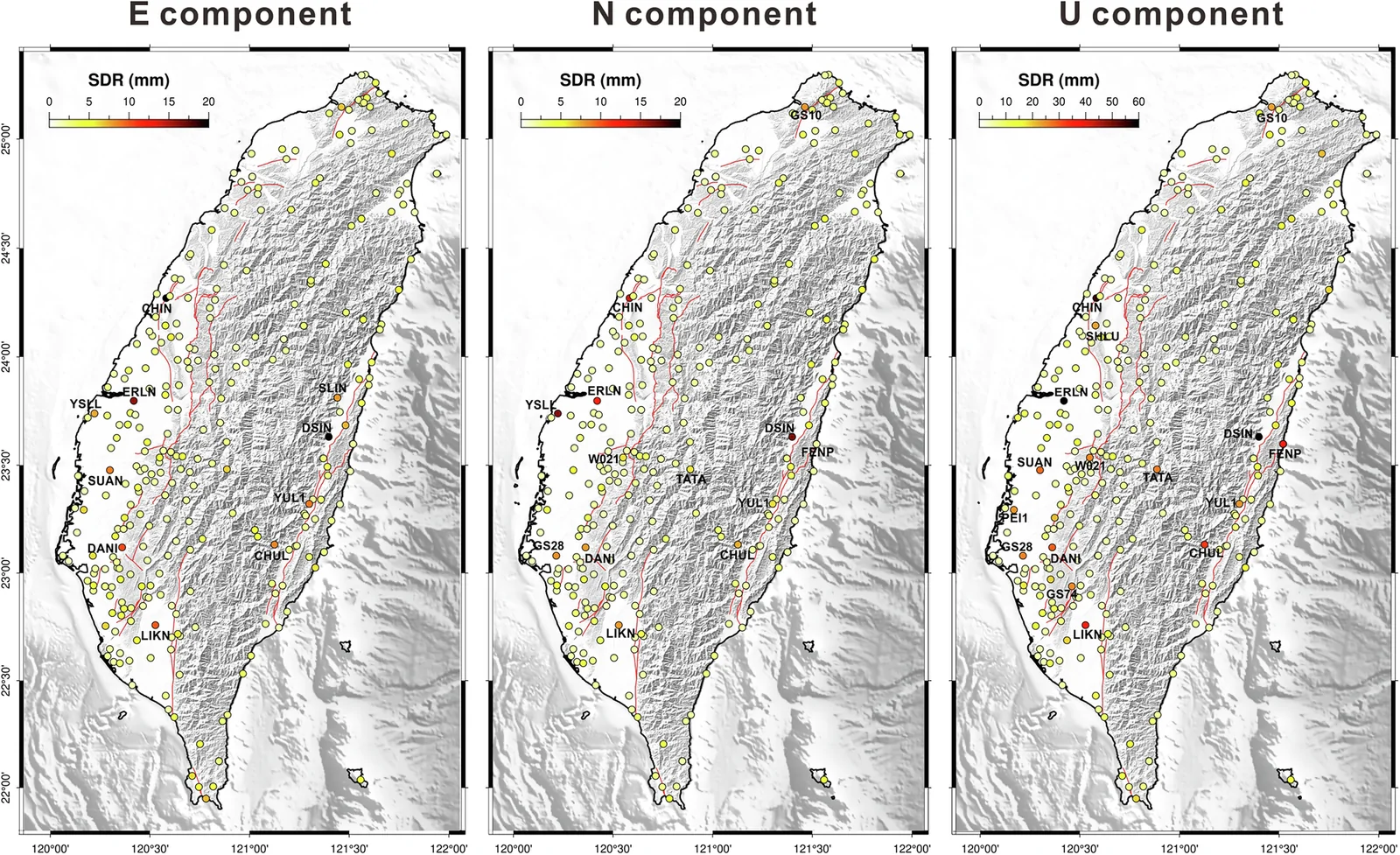
Spatial distributions of SDR values for cGNSS stations across Taiwan. Panels (**a**), (**b**), and (**c**) show SDR values for the east (E), north (N), and vertical (U) components, respectively. The color scale represents SDR magnitude. Stations with SDR values exceeding the quality-control threshold (median +2σ) are annotated on the maps.

Quality-control thresholds were defined as the median SDR plus two standard deviations for each component, corresponding to 7.3 mm for the E component, 5.8 mm for the N component, and 21.4 mm for the U component. Figure 4 presents the spatial distributions of SDR values across Taiwan. A total of 10, 13, and 16 stations exceeded the defined thresholds in the E, N, and U components, respectively. These stations are summarized in Supplementary Table S1 and annotated in Fig. 4 for reference.

Stations exhibiting elevated vertical SDR values are often located at sites with reduced satellite visibility, reflecting the impact of environmental obstructions and multipath effects on time-series stability.

Overall, most cGNSS stations in Taiwan exhibit stable long-term performance suitable for geodetic applications. The combined evaluation of data availability, satellite visibility, and SDR provides an objective framework for identifying stations and time periods potentially affected by degraded observation conditions.

## Usage Notes

The archived dataset provides consistently GNSS coordinate time series and associated quality metrics that support a wide range of geodetic applications in Taiwan.

The time series can be used to estimate interseismic deformation and crustal velocity fields over different time periods following standard preprocessing procedures, including removal of offsets and seasonal signals. The dense station distribution also enables investigations of coseismic and postseismic deformation associated with large earthquakes and supports comparisons with other geodetic and seismological observations.

Beyond tectonic applications, the dataset is suitable for studies of slow slip events, hydrological loading, land subsidence, and other environmental and surface processes. The availability of consistently processed products and station-level quality indicators facilitates consistent analyses across the network and supports integration with complementary geodetic and remote sensing datasets.

Because the archive includes both primary daily PPP coordinate solutions and derived corrected products, users may either adopt the provided offset-corrected and seasonally filtered time series or perform their own time-series analysis from the primary daily PPP solutions. This flexibility is important because choices in outlier screening, discontinuity detection, stochastic noise modelling, and velocity estimation may depend on the scientific application.

## Supplementary information

Supplementary Table S1

## Data Availability

The dataset described in this study is available from Zenodo at https://doi.org/10.5281/zenodo.20047819. The archived release covers the period 2010–2024 and includes daily GNSS position time series, station metadata, and quality metrics derived from time-series analysis. Raw GNSS observation data are maintained 398 by the respective operating agencies and are not redistributed as part of this dataset.

## Code availability

GPS data were processed using the GipsyX/RTGx software package (version 2.5; https://gipsy-oasis.jpl.nasa.gov/). The Generic Mapping Tools (GMT, version 6.4; https://www.generic-mapping-tools.org/) were used for visualization. Custom MATLAB scripts used for GNSS time-series modeling and correction are publicly available at https://github.com/cuzn1103/taiwan-gnss-timeseries-correction. The code implements the time-series modelling and correction procedures described in this study, including outlier removal and estimation of linear and seasonal components. Some processing steps, such as satellite visibility analysis, rely on auxiliary files and intermediate outputs generated during GNSS processing (e.g., observation tracking information) and are therefore not fully included in the code repository. The resulting quality metrics used in this study are provided in the archived dataset, and the corresponding procedures are described in the Methods section.
